# Atomic Force Microscopy Probing and Analysis of Polyimide Supramolecular Systems for Sensor Devices

**DOI:** 10.3390/s23094489

**Published:** 2023-05-05

**Authors:** Iuliana Stoica, Andreea Irina Barzic, Cristian Ursu, George Stoian, Elena Gabriela Hitruc, Ion Sava

**Affiliations:** 1 “Petru Poni” Institute of Macromolecular Chemistry, 700487 Iasi, Romania; stoica_iuliana@icmpp.ro (I.S.); irina_cosutchi@yahoo.com (A.I.B.); cristian.ursu@icmpp.ro (C.U.); gabihit@icmpp.ro (E.G.H.); 2 National Institute of Research and Development for Technical Physics, 700050 Iasi, Romania; gstoian@phys-iasi.ro

**Keywords:** polyimide supramolecular system, azochromophore, flexible support, laser irradiation, surface relief gratings, texture characterization, tip modification, adhesion, wettability, interface

## Abstract

A series of polyimide supramolecular systems containing different amounts of azochromophore were tested as flexible supports that can be used in the fabrication of certain devices, such as sensors for monitoring the temperature changes, by coating them with conductive metals. That is why it is required to have good interfacial compatibility between the flexible substrate and the inorganic layer. The interface of the sensor elements must be designed in such a way as to improve the sensitivity, accuracy, and response time of the device. Laser irradiation is one of the commonly employed techniques used for surface adaptation by patterning polyimides to increase contact and enhance device reliability and signal transmission. In this context, this work highlights unreported aspects arising from the azo-polyimide morphology, local nanomechanical properties and wettability, which are impacting the compatibility with silver. The texture parameters indicate an improvement of the modulations’ quality arising after laser irradiation through the phase mask, increasing the bearing capacity, fluid retention, and surface anisotropy when the amount of the azochromophore increases. The force curve spectroscopy and wettability studies indicated that the modification of the polymer morphology and surface chemistry lead to a better interfacial interaction with the metal lines when the azo component and the polyamidic acid are in equimolar quantities.

## 1. Introduction

In the latest years, a remarkable evolution of electronic products has been observed under the trend of miniaturization and improvement of the properties of some components implemented in the devices [1]. Special attention was ascribed to sensor fabrication with the purpose of making them suitable for more practical uses [2]. Flexible supports were found to introduce many benefits for the production of sensors, namely, they reduce expenses and enable wider area of manufacturing, concomitantly with thinner and denser packages [3]. These features are expanding the path into the market of such devices [4]. In contrast to classic counterparts (like silicon or glass), many polymer materials have the ability to optimize the device’s contact with a non-planar surface because they are displaying stretchability, rollability, and bendability properties [5,6,7]. The perspectives introduced by flexible/printed electronic sensors attracted tremendous focus on the development of novel materials and fabrication approaches [8,9]. It is worth noting that electronics industry ordinarily tests as flexible substrates several kinds of polymers, including polyethylene terephthalate [10], cellulose [11], polyethylene naphthalate [12], polyvinyl alcohol [13], polyurethane [14], polyaniline [15], polydimethylsiloxane [16], and polyimide (PI) [17]. The latter type of material is broadly recognized because of its stability to elevated temperature, electrochemical resistance, mechanical strength, and electrical insulation [18,19]. The ability of PIs to keep their performance during the action of high temperatures, they are preferred for making sensors of temperature [20].

Polyimides are known to have good adhesion to metal coatings which renders elevated level of strain delocalization in a wide temperature interval. As a result, deposition procedures, such as sputtering and e-beam evaporation, are recommendable for this sort of flexible polymer support [21]. There are few developments in flexible temperature sensors made on polyimide supports. Xiao and co-workers [22] made a temperature-sensing device by depositing it on a commercial polyimide which was patterned by image reversal photolithography to include the platinum sensor. Their report indicated that better device sensitivity and response frequency are accomplished when the parasitic heating losses are lowered. Chia et al. [23] described the path to obtain such a sensor by a “double sided” manufacturing approach, where a platinum serpentine was deposited on a polyimide. Dankoco and collaborators [24] have designed a thermistor made of silver casted on a commercial polyimide tape and the conductive lines were patterned via inkjet printing technology. Gandla et al. [25] showed another way to build a sensor via laser-generated carbonization on a polyimide foil on top of which is introduced a printed circuit. Al Hashimi et al. [26] employed photolithography to attain a sensor that solves the issues of adhesion found in the Pt-based products. Thus, they propose to use a layer of nickel with copper leads which is placed on a polyimide.

The most investigated sensors that are monitoring temperature changes are the resistive detectors. They are operating by recording the electrical signal modification of the thermosensitive layer under exposure to variable heating [24]. Generally, the fabrication of these devices involve the use of flexible polymer (i.e., polyimide) which is coated with conductive metals, such as gold [27], platinum [23], or silver [28]. For this reason, it is imperative that flexible support must exhibit good interfacial compatibility with the inorganic layer. The quality of the contact between the polymer component and metallic lines is influencing the sensitivity, accuracy, and response time of the device. Therefore, a great level of sensitivity is accomplished as a function of the design of the interface of the sensor elements [29]. In this context, the use of patterned polymer substrates to attain flexible temperature sensors has gained increasing importance [30]. Modification of the support surface to increase the contact area is desirable to enhance the device’s reliability and signal transmission. Among the commonly employed techniques used for surface adaptation of the polymers to facilitate their compatibility with metals, the most relevant ones are plasma etching [31], chemical treatments [32], and laser irradiation [33]. Such approaches produce modifications of the polymer morphology and surface chemistry that lead to better interfacial interaction with the metal lines. Laser exposure of polymers seems to be advantageous because it allows spatial resolution of the surface features [34].

The surface characteristics of the polymer support are essential for the performance of sensors. It is known that the surface features (morphology, roughness, and polarity) of the polymers are crucial for establishing how to acquire good adhesion to metals [35,36,37,38]. However, these aspects are not deeply exploited in the reports on temperature sensors. Nuzhdin et al. [39] have analyzed the operability of a sensing device on diffraction microstructures represented by polymers with metal nanoparticles. Yu et al. [40] demonstrated that the surface roughness of the support is leading to the occurrence of micrometric cracks in the sensing film which yields raised sensitivity. However, none of these studies refer to polyimides.

In our previous works [41,42,43,44], we reported that we can adapt the morphology of polyimides via laser irradiation and distinct surface characteristics are attained as a function of polymer structure and exposure conditions. Moreover, when associating a polyimide with an azochomophore, laser treatment leads to surface texture in the form of surface relief gratings (SRG) having the distance between them induced by the phase mask pitch [41,42,43,44]. This work highlights unreported aspects arising from the polyimide morphology, local mechanical properties, and wettability which are impacting the compatibility with silver. The flexible supports proposed here are non-commercial polyimides which can be easily shaped via laser irradiation due to the incorporation of variable amounts of azo dye.

## 2. Materials and Methods

### 2.1. Materials

**Scheme 1:**
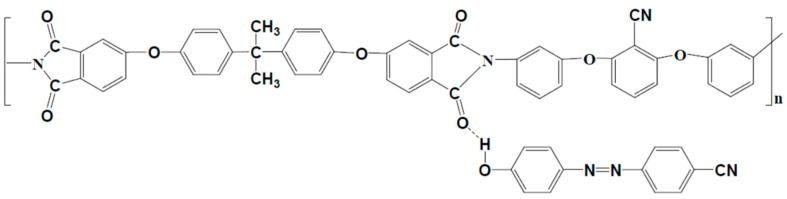
Chemical structure of the studied polyimide supramolecular system.

The polyimide supramolecular systems were realized by introducing the 4-[(4-cyanophenyl)diazenyl]phenol in different molar ratios into the solution of polyamidic acid obtained by polycondensation reaction of 2,6-bis(3-aminophenoxy) benzonitrile and 4,4′-isopropylidene-diphenoxy-bis(phthalic anhydride) in equimolar quantities. The molar ratio between the azochromophore and the polyamidic acid was 0.25:1, 0.5:1, 0.75:1, and. 1:1, resulting after casting onto glass plates and thermal treatment (50 °C for 4 h, 100 °C for 1 h, 125 °C for 1 h, 150 °C for 1 h, 175 °C for 1 h, and 200 °C for 1 h) the following azo polyimide supramolecular systems Az_0.25_PSS, Az_0.50_PSS, Az_0.75_PSS, and Az_1.00_PSS, respectively, in the form of flexible films. The steeping thermal treatment (called thermal imidization process) was mandatory to realize the stable final structure of polyimide [45]. Otherwise, the supramolecular polyimide system is no longer formed, remaining at the azodye/polyamidic acid stage. The synthesis and complete characterization of these compounds was previously reported in [46]. The final chemical structure of the polyimide supramolecular system is presented in Scheme 1:

### 2.2. Methods

Permanent surface relief gratings were created on azo-polyimide films with different compositions by using the near-field interference pattern generated when a pulsed UV laser beam was incident on a diffraction grating. The third harmonic of a Nd:YAG laser (Brilliant, Quantel; 1064 nm, 10 ns pulse length, 6 mm in diameter) incident on a 3× Galilean expander was projected through a phase mask of 1000 grooves/mm (Edmund optics). A schematic representation of the used experimental arrangement was previously presented in Figure 1 from [46]. A 1 mm-thick quartz plate was positioned between the phase mask and polymer film, and two additional quartz plates were positioned at the lateral sides of the system to prevent contamination of the phase mask and ensure correct handling of the system. Permanent relief gratings with the same periodicity as that of the phase mask were generated on PI-based supramolecular systems through irradiation sequences performed at a constant laser fluence of 45 mJ/cm^2^, a total delivered laser pulses of 300, and a pulse repetition frequency of 10 Hz. The subsequent labels: Az_0.25_PSS-L, Az_0.50_PSS-L, Az_0.75_PSS-L, and Az_1.00_PSS-L were attributed to the laser-irradiated films.

Atomic force microscopy (AFM) measurements were made using Nova 1.1.1.19891 software, through which the NTEGRA multifunctional Scanning Probe Microscope (NT-MDT Spectrum Instruments, Zelenograd, Moscow, Russia) was manipulated in atmospheric conditions at 23 °C. The cantilever type used for the experiments was NSG03 (length = 150 ± 10 µm, width = 24 ± 7 µm, thickness = 1.5 ± 0.75µm, tetrahedral shape tip curvature radius= 6–10 nm) from TipsNano OÜ, Tallinn, Estonia. The resonant frequency of the cantilever was 88 kHz. The topographical images were obtained on different scan sizes, in tapping mode, simultaneously with the phase contrast and amplitude images. The local mechanical properties, namely the mean adhesion force (Fadh), were estimated from 9 force curves. A force curve is the representation of the cantilever’s deflection (DFL signal) vs. the tip–sample distance during the approach-retraction maneuver. That is why it is also named DFL-height curve. The DFL-height curves were acquired through the AFM Force Curve Spectroscopy (FCS), in contact mode, on the collected morphological images. The adhesion force was deduced from the force curve according to Hooke’s law: Fadh = −k∙Δx where k is the force constant of the cantilever and Δx is the deviation of the cantilever reported to the sample, taking the maximum pulling force at the final contact state before the jump of the tip from contact during retraction. MountainsSPIP^®^ Academic (2023) surface analysis software by DigitalSurf (Besançon, France) was used to evaluate the texture characteristics.

To evaluate the interaction of silver with the irradiated samples, thin layers of silver were deposited on NSG03 cantilevers by the sputtering method, using the K550X Sputter Coater system that uses a Magnetron Target Assembly. The separation distance between the silver sputtering target (cathode) and the anode, where the cantilevers were installed during deposition (with the exposed side where the tip was located) was 9 cm. The voltage was 900 V and the current was 10 mA. The sputtering process was made using air as the working gas, under a pressure of 60 Pa, for 5, 10, and 15 min. This device does not allow connection to cylinders containing other types of gases, such as argon or any other inert gas. It is estimated that only 5% of the deposed silver is silver oxide, the rest being metallic silver (these data belong to an unpublished work).

The images of the pristine and modified tips have been acquired through scanning electron microscopy (SEM) using a Crossbeam System Neon 40ESB FIB/SEM microscope from Karl Zeiss AG (Oberkochen, Germany) with thermal Schottky field emission, equipped with EDS, In-lens, SE, and EsB detectors. Electron beam resolution: 1.1 ÷ 2.5 nm for U = 20 ÷ 1 kV. For acquiring the SEM images, we used the In-lens and SE detectors under the following conditions: electrons accelerating voltages (EHT) of 1.8 and 20 kV and a working distance (WD) of around 5 mm. For the EDX analysis, an X-Max 50 detector along with its dedicated software (INCA) from Oxford Instruments Analytical Ltd. (High Wycombe, Buckingamshire, UK) were used with electrons accelerating voltage of 20 kV and a working distance of 5.1 mm.

The test grating, TGT1 (NT-MDT Co, Zelenograd, Moscow, Russia), formed from an assembling of sharp tips placed on Si wafer surface (height = 0.3–0.5 μm, tip angle = 50 ± 10°, and tip curvature radius = 10 nm) was used to visualize and to recreate the modified tips.

The contact angle tests were made to evaluate the changes in the surface polarity of the polyimide supramolecular systems. Water and formamide were utilized as test liquids and the measurements were performed five times on a lab made device. The instrument was designed to contain a video recording part to visualize the size/shape of the drops placed on the sample surface with a Hamilton syringe. The experiments were performed in temperature-controlled conditions. More details can be found in another report [47]. The measuring error of the contact angle was ±1°.

## 3. Results and Discussion

### 3.1. Evaluation of the Texture Modifications through UV-Laser Irradiation

Surface texturing is particularly important in polyimide applications as flexible support for depositing electrodes. A good anisotropy of the surface will, thus, improve the final performance of the applied flexible sensors. That is why a complete and complex characterization of the surface morphology is crucial in order to identify the surface characteristics and how they subsequently influence the deposition of the metal, namely silver, in order to print the sensor. Polyimide supramolecular systems, having an azo component in their structure, lend themselves to texturing by laser irradiation, at a wavelength of 355 nm, demonstrated by other previous studies [41,42,43,44] to be suitable for stimulating the creation of SRGs on the top of the samples. An atomic force microscopy is a versatile tool that can be used to evaluate the evolution of the surface morphology following laser irradiation in tapping mode without damaging the resulting texture. Both the amplitude and phase images were achieved concomitant with the topography in tapping mode (also called intermittent contact, or semi-contact mode). The topography and amplitude images were acquired in the forward scanning direction, while phase images were obtained in the backward scanning direction. The phase imaging mode images provide information about the nanomechanical properties of the sample surface (stiffness, adhesion, friction, viscoelasticity, etc.). In the phase imaging mode, the comportment of the cantilever’s tip that periodically comes into contact with the sample surface, is influenced by a different type of forces, such as adhesive, repulsive, capillary, and so on, resulting in some shift of the phase. Assuming that the investigated surface shows inhomogeneities and a certain distribution of its characteristics, they can be highlighted through the phase shift distribution over the specimen surface. On the other hand, the semi-contact error mode images (amplitude images) are frequently used to identify small aspects that can be found on the relief background, highlighting the shape of the surface (the borders of the features) and complementing the information derived from the height image. In the semi-contact error mode, throughout the time of scanning, the feedback loop cannot immediately balance the modifications of the Mag signal (which is related to the cantilever oscillation amplitude), having a time retardation. Thus, the current value of the Mag signal (expressed in nA) is the error signal of the feedback loop and, if it is registered, offers supplementary and more detailed information on surface topography.

**Figure 1:**
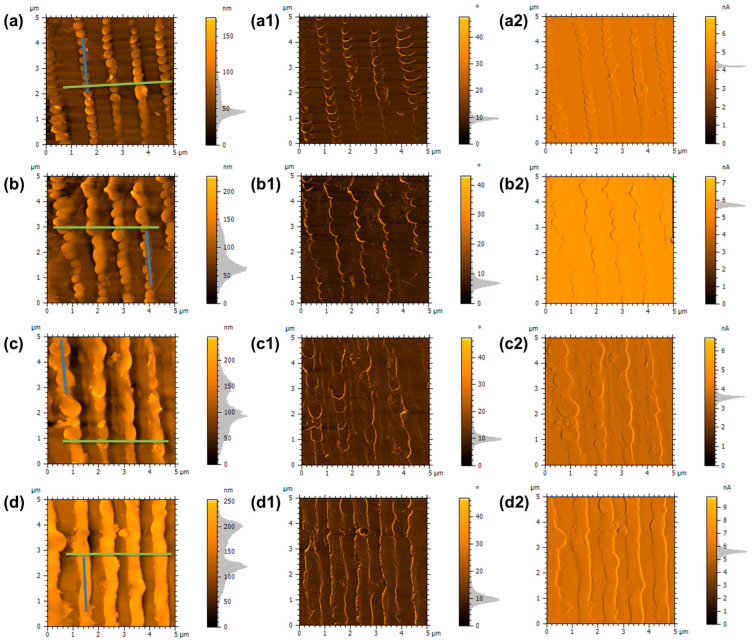
Simultaneous AFM topography, phase contrast, and amplitude images performed on phase mask laser irradiated Az_0.25_PSS-L (**a**,**a1**,**a2**), Az_0.50_PSS-L (**b**,**b1**,**b2**), Az_0.75_PSS-L (**c**,**c1**,**c2**), and Az_1.00_PSS-L (**d**,**d1**,**d2**). The positions of the cross-section profiles taken perpendicularly to the SRGs are marked with green lines, while the ones along the SRGs are marked with blue lines. In the amplitude images, nA is a procedure-defined unit.

**Figure 2:**
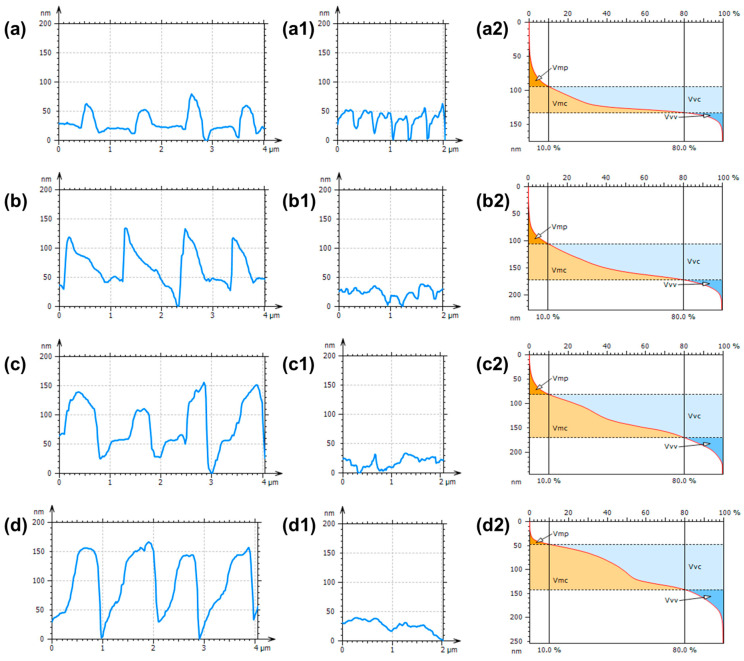
Cross-section profiles taken perpendicularly and along the SRGs and Abbott curves used to calculate the functional volume parameters, based on the AFM topography images, obtained for the phase mask laser irradiated Az_0.25_PSS-L (**a**,**a1**,**a2**), Az_0.50_PSS-L (**b**,**b1**,**b2**), Az_0.75_PSS-L (**c**,**c1**,**c2**), and Az_1.00_PSS-L (**d**,**d1**,**d2**).

In Figure 1 are presented the processed height, phase contrast, and amplitude AFM images collected from phase mask laser irradiated Az_0.25_PSS-L (a,a1,a2), Az_0.50_PSS-L (b,b1,b2), Az_0.75_PSS-L (c,c1,c2), and Az_1.00_PSS-L (d,d1,d2) samples. Analyzing these images, it is found that the morphology after the irradiation process is influenced by the amount of azochromophore introduced into the system. When the molar ratio between azodye and polyamidic acid was 0.25:1, the formation of a repetitive texture, composed of SRGs, was observed. According to the cross-section profiles taken perpendicularly and along the SRGs from Figure 2a,a1) and the data in Table 1, they have an average height of around 50 nm and seem to be formed by the close self-assembled spherical nanostructures. This aspect indicates that predominantly in the formation of these supplementary nano-modulations along the vertical direction is the photo-fluidization process [43,48]. As the amount of azochromophore in the system increases, the self-assembled spherical nanostructures tend to join more and more, until at the equimolar ratio where they disappear, and the resulting SRGs having a uniform appearance. The height of the SRGs, thus, increases to 90 nm for the molar ratio 0.5:1 (Figure 2b), 125 nm for the molar ratio 0.75:1 (Figure 2c), and finally reaching around 160 nm for Az_1.00_PSS-L (Figure 2d). The uniformity along the modulations was confirmed by the aspect of the amplitude images (Figure 1a2,b2,c2,d2 where nA represents a procedure-defined unit) and by the average roughness, Ra, displayed in Table 1. This parameter was calculated from the cross-section profiles (Figure 2a1,b1,c1,d1) taken on top of SRGs, along the ridge, as indicated by the blue lines from Figure 1a,b,c,d. As the evenness increases, Ra decreases significantly, reaching 3 nm for the sample Az_1.00_PSS-L. The modulation heights influenced the total Sq of the scanning area, increasing with their increase, up to around 45 nm (Table 1). The phase contrast images show that the phase shift is greater at the border (middle slope) of the formed structures (the brighter regions in Figure 1a1,b1,c1,d1), once again supporting the discoveries made in previous studies [42], according to which in these regions, Young’s modulus is lower and the deformation is higher than in the other regions, where these characteristics are constant. Since the shift of the phase is influenced by the characteristics of the investigated material (differences in hardness and softness) and by the repulsive, adhesive, capillary, and other forces, this correlation can be achieved, even if the measurements are based on different principles.

**Figure 3:**
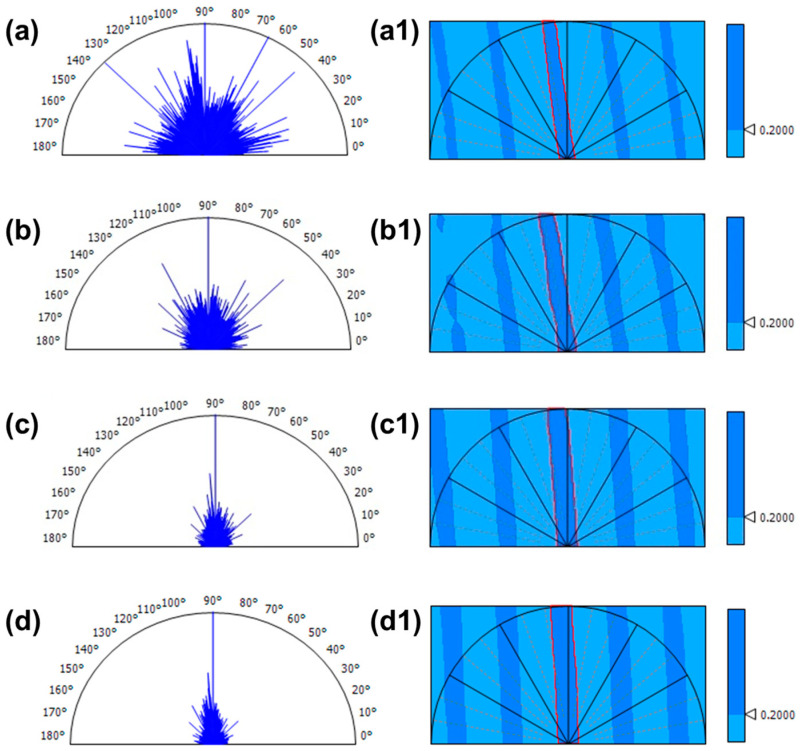
Texture direction representation (based on the Fourier Transform) and texture isotropy representation (based on the autocorrelation function) supported by the AFM topography images, obtained for the phase mask laser, irradiated Az_0.25_PSS-L (**a**,**a1**), Az_0.50_PSS-L (**b**,**b1**), Az_0.75_PSS-L (**c**,**c1**), and Az_1.00_PSS-L (**d**,**d1**). The angles are measured anticlockwise. Same as in texture direction representation, in the texture isotropy representation, the black lines indicates the positions of the main angle lines (0°, 30°, 60°, 90°, 120°, 150°, 180°), the dotted orange lines indicates the positions of the intermediary angle lines and the red lines delimits the periodic motif.

Figure 3 displayed the texture direction representations (based on the Fourier Transform) for the phase mask laser irradiated Az_0.25_PSS-L (a), Az_0.50_PSS-L (b), Az_0.75_PSS-L (c), and Az_1.00_PSS-L (d). By means of these polar representations, it was highlighted that the appearance of additional hierarchical structures (sample Az_0.25_PSS-L) implies a weak anisotropy of the morphology indicated by a lower value but close to 0.500 on the texture direction index, Stdi (Table 1). As the SRGs are better defined and become unidirectional, the texture direction representation also evolves towards a single direction, a fact confirmed by the increasingly smaller value of Stdi which finally reaches close to 0.200 (Table 1). Based on the texture isotropy representation (built on the autocorrelation function) supported by the AFM topography images, obtained for Az_0.25_PSS-L (Figure 3a1), Az_0.50_PSS-L (Figure 3b1), Az_0.75_PSS-L (Figure 3c1), and Az_1.00_PSS-L (Figure 3d1), it was found that the periodicity of the SRGs also increased up to almost 70% with the increase in the amount of the azo component (Table 1), and their period was dictated by the characteristics of the phase mask (reaching approximately one micrometer for the best structured sample).

**Figure A1:**
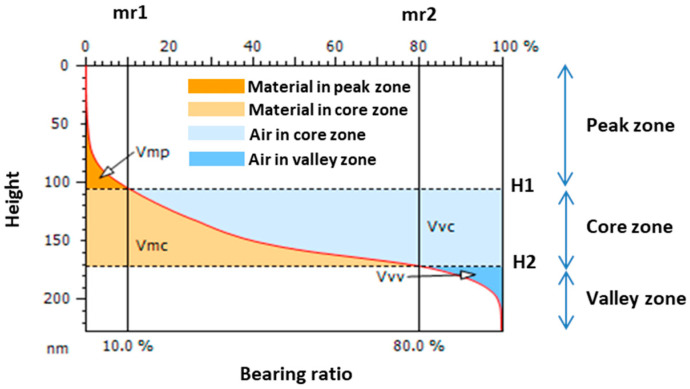
Representative Abbott curve illustrating the three created zones: the peak region, the core region and the valley region and the calculation of the corresponding functional volume parameters Vmp, Vmc, Vvc and Vvv.

Regarding the functionality of the irradiated surfaces for the applications taken under consideration, the functional index (Sbi: surface bearing index) and functional volume parameters (Vmp: peak material volume; Vmc: core material volume; Vvc: core void volume; Vvv: valley void volume) (Table 2) were evaluated from the Abbott curves (Figure 2a2,b2,c2,d2). A brief introduction to the methodology of Abbott curves, including the definition of the mentioned functional index and functional volume parameters, is presented in Appendix A (Figure A1). The general appearance of the Abbott curves was also influenced by the amount of azoderivative used in the synthesis of polyimide supramolecular systems. The bearing capacity (Sbi) increases due to the evolution of peak and core material volume with the increase of the molar ratio between the azo part and the polyamidic acid, a favorable fact for the tracked applications. Moreover, the increase of the core and valley void volume can improve, for example, the ink retention based on silver nanoparticles in the core and valley regions of the relief.

### 3.2. Local Adhesion Studies via Silver Modified Tips

**Figure 4:**
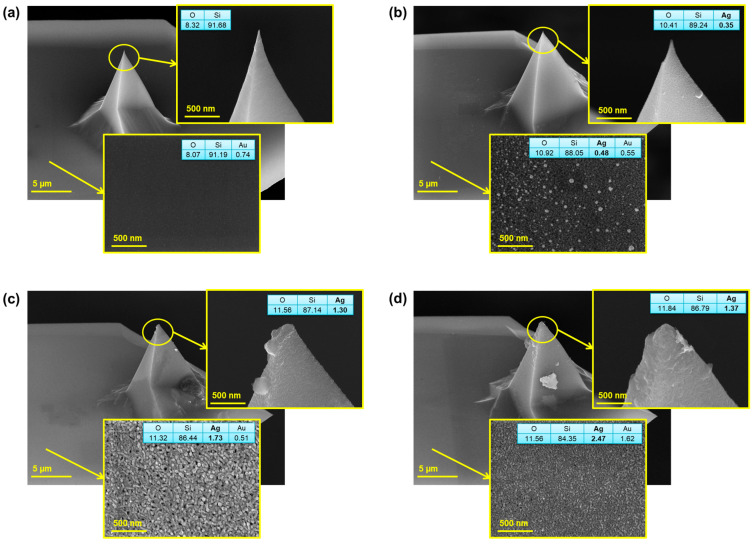
SEM images of the unmodified (**a**) and silver sputtered NSG03 AFM cantilevers for 5 min (**b**), 10 min (**c**), and 15 min (**d**), respectively.

In order to evaluate how silver would adhere to polyimide supramolecular systems, having different amounts of the azo component, irradiated with a UV laser, we tried to obtain tips/cantilevers uniformly covered with silver. Such tips compatible with the supplied atomic force microscope are not commercially available, therefore, they had to be manufactured in the laboratory through a sputtering process. NSG03-type cantilevers were used, and the deposition time was varied from 5 min to 10 min and 15 min, respectively. The monitoring of the result of the silver deposition process both on the cantilever in its ensemble, and on the tip, was carried out by means of SEM images and EDX analysis. Thus, Figure 4 presented the SEM images of the unmodified NSG03 cantilever (a) and silver sputtered NSG03 AFM cantilevers for 5 min (b), 10 min (c), and 15 min (d). By means of this method it can be observed that for short deposition times, the silver nanoparticles are at a certain distance from each other, leaving the silicon-free space to be exposed. As the silver deposition time increases, the nanoparticles better cover both the base of the cantilever and its tip, until after 15 min, they are so dense that they form a uniformly deposited layer without exposed areas of the initial tip. This fact is also confirmed by the percentages of silver in the EDX analysis.

**Figure 5:**
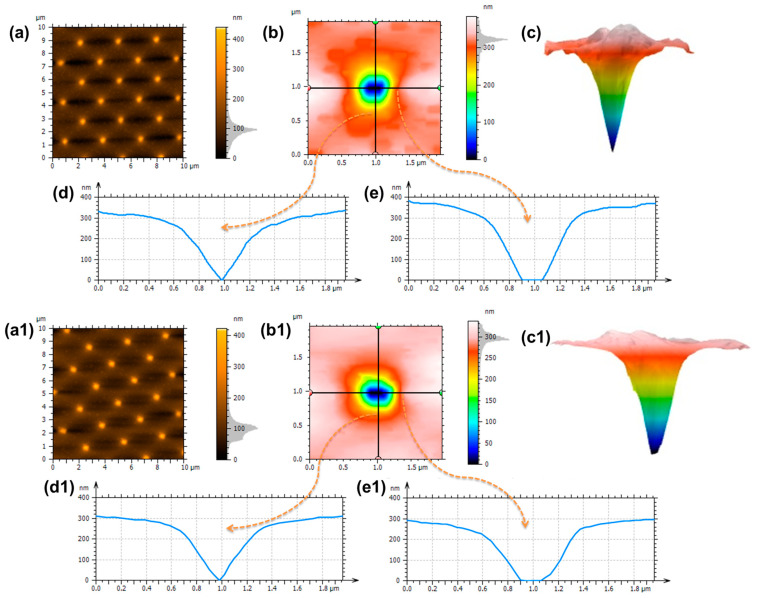
The blind reconstruction of the uncovered NSG03 tip (**a**,**b**,**c**,**d**,**e**) and 15 min silver sputtered NSG03 tip (**a1**,**b1**,**c1**,**d1**,**e1**): AFM images of the TGT1 test grating (**a**,**a1**), 2D perspectives of the reconstructed tips (**b**,**b1**), 3D perspectives of the reconstructed tips (**c**,**c1**), tips X-profiles (**d**,**d1**), and tips Y-profiles (**e**,**e1**).

Since the NSG03 AFM cantilever subject to the sputtering process for 15 min gave the best results in terms of silver coating, this cantilever was further used to test the local nanomechanical properties, namely the adhesion force between the textured polyimide substrates and silver. For this purpose, first the MountainsSPIP^®^ Academic software was used to visualize and characterize the sharpness, deterioration, and impurification of the 15 min silver sputtered NSG03 tip and, for comparison, of the uncovered NSG03 tip based on a 10 × 10 μm^2^ AFM scan of the reference TGT1 test grating (Figure 5a—uncovered NSG03 tip and Figure 5a1—15 min silver sputtered NSG03 tip). Thus, the blind reconstruction method was applied, similar to the cases described in the literature [49,50,51,52], and the 2D and 3D perspectives of the reconstructed tips (Figure 5b,c,b1,c1) were obtained by deconvolution of the test grating AFM images. From the tips X-cross-section profiles (Figure 5d,d1) and tip Y-cross-section profiles (Figure 5e,e1) taken as indicated in the blind reconstruction images, the average uncovered NSG03 tip radius established by a sphere fit to the evaluated tip, was 11 ± 2 nm, while the radius of the silver covered tip was 65 ± 5 nm. These values were confirmed by the SEM measurements. These data prove once again that the tip subject to the sputtering process for 15 min is indeed modified.

After the tip reconstruction process, some quantitative results were extracted, namely the resonance frequency of 101 kHz and the cantilever’s normal spring constant of 3.5 N/m, determined by John Sader’s method [53,54], using data on the resonance peak and planar dimensions of cantilever (length = 135 µm, width = 30 µm). Prior the adhesion force measurements, it was necessary to rescale the values of DFL (deflection of the cantilever) from relative units (nA) to the units of physical force (nN), taking into account the sensitivity of the optical system ($\frac{∆Z}{∆DFL}$), describing the slope of the left side of an approach curve, the cantilever’s normal spring constant (k), the DFL value expressed in relative units, nA (${DFL}_{nA}$), and the value of the DFL signal far from the surface given in nA (${DFL}_{0}$), according to the following relation:

(1) $${DFL}_{nN}=\frac{∆Z}{∆DFL}\times k\times \left({DFL}_{nA}-{DFL}_{0}\right)$$

**Figure 6:**
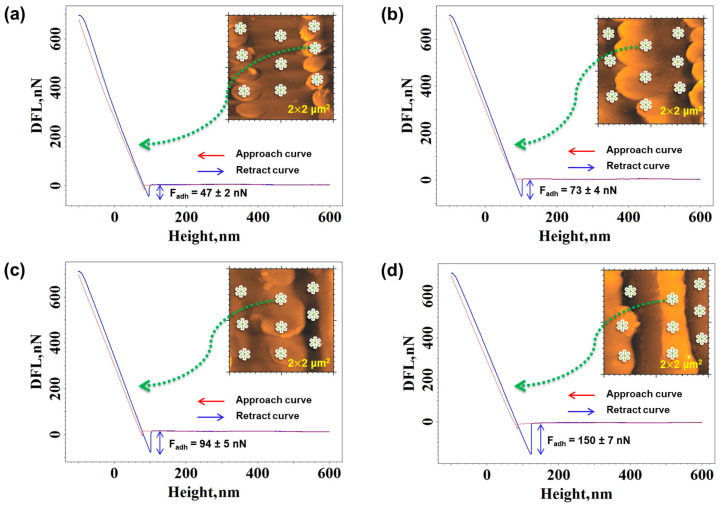
Representative DFL-height curves collected from 2 × 2 μm^2^ AFM topography images recorded for the phase mask laser irradiated Az_0.25_PSS-L (**a**), Az_0.50_PSS-L (**b**), Az_0.75_PSS-L (**c**) and Az_1.00_PSS-L (**d**). The red curves are the approach curves. The blue curves are the retract curves, from where the average adhesion force (Fadh) was calculated.

Once this calibration was conducted, force curves were collected using a scan size with the side of 2 μm for Az_0.25_PSS-L (Figure 6a), Az_0.50_PSS-L (Figure 6b), Az_0.75_PSS-L (Figure 6c), and Az_1.00_PSS-L (Figure 6d), in the places indicated by the asterisk symbol (*). As seen in this Figure 6, the force curves were taken both in the exposed and unexposed regions through the phase mask. According to our previous study [42], where the nanomechanical measurements were made on a Park NX10 Atomic Force Microscope, using PinPoint Nanomechanical mode, with a very good resolution, the value of the adhesion force measured on the top hill (regions covered by the phase mask, unexposed to the laser beam), and bottom valley and base line of the SRGs (regions uncovered by the phase mask, exposed to the laser beam) does not show significant variations. That is why the locations indicated by asterisks were chosen. Only representative force curves were presented in Figure 6. From the retract curves, the average adhesion force (Fadh) was calculated for each sample as the highest extraction force at the final contact position preliminary to the leap of the tip from contact with the surface of the specimen.

Analyzing the values in Figure 6, it can be seen that the adhesion strength of silver with the polyimide samples, irradiated under the same conditions, increases with the increase in the amount of azochromophore in the system. Thus, the lowest value of the silver/polyimide adhesion force was obtained for the molar ratio between the azochromophore and the polyamidic acid of 0.25:1, and the highest value for 1:1, the sample for which the best repetitive texturing of all was obtained. This indicates that the sample Az_1.00_PSS-L is the most suitable for depositing the silver electrodes in order to obtain the sensor.

### 3.3. Interfacial Adhesion Estimation

The study of the surface tension of the polyimide support is very important for evaluating the interaction of the samples with other materials. The Fowkes method [55] permits the determination of the surface tension parameters of the examined films by considering the geometric mean contributions of interactions and that the overall surface energy represents the sum of all forces at the interface. These aspects are expressed by Equations (2) and (3):

(2) $$1+cos\theta =\frac{2}{\sigma_{l}}\sqrt{\sigma_{l}^{p}\sigma_{s}^{p}}+\frac{2}{\sigma_{l}}\sqrt{\sigma_{l}^{d}\sigma_{s}^{d}}$$

(3) $$\sigma_{s}=\sigma_{s}^{p}+\sigma_{s}^{d}$$

where *θ* is the contact angle, *σ* is the surface tension, the subscripts “*l*” and “*s*” refer to the liquid and the solid phase, and the indices “*p*” and “*d*” reveal the polar and dispersive part of *σ*. The contact angle data attained with the chosen test liquids casted on the film samples are summarized in Table 3. The magnitude of water contact angle seems to reduce as the content of azo dye is increasing. This is possibly due to the formation of more polar hydrogen bonding among the system components. After the laser irradiation, the surface properties are changed differently as a result of variable sensitivity of samples to molecular ordering under polarized radiation action. Thus, as the amount of azo dye increased in the system, laser irradiation of the samples determined the decrease of the contact angle. This result is supported by the literature [56] which shows (for other type of polymers) that laser structured films display lower values of the contact angle in comparison to the pristine ones. Based on the recorded contact angle, the surface tension components were computed and listed in Table 3. The measuring error of the contact angle was ±1° and this was reflected to the second decimal of the surface tension data. It can be noted that the surface polar character is enhanced by addition of the azo component. This can be caused by formation of more hydrogen bonds which are producing powerful dipole–dipole interactions among the constituent molecules in the samples. At the same time, the dispersive forces are not ranging very much with the sample composition. Laser irradiation of the polyimide films determines a slight reduction of the $\sigma_{s}^{d}$, while $\sigma_{s}^{p}$ is increasing by inserting azo-component quantity. The balance between the dispersive and polar components of the surface tension is influencing the ability of interaction of samples with metal. The work of adhesion with silver ($W_{Ad}$) was estimated using the relation (4):

(4) $$W_{Ad}=2\sqrt{\sigma_{Ag}^{p}\sigma_{s}^{p}}+2\sqrt{\sigma_{Ag}^{d}\sigma_{s}^{d}}$$

According to Table 3, the increase in the polymer surface polarity is reflected in an increase of the adhesion interactions of the samples with silver. Moreover, as the laser exposure changes even more $\sigma_{s}^{p}$, it seems that this was conducted to further increase the adhesion forces of surface textured samples in regard to the unexposed ones. Such aspects are favorable for sensor fabrication because they contribute to better reliability of the device.

Thus, in this work, the adhesion properties were evaluated by two different methods at the macroscale and at the nanoscale. The apparent discrepancies between the values of the adhesion derive from the following aspects. First, the adhesion force (expressed in nN) is defined as the force necessary to retract the cantilever’s tip from the sample surface. Consequently, the adhesion force relies on the local nanoscale interaction between the silver-modified cantilever’s tip and the sample surface. Then, the work of adhesion (expressed in mN/m) is defined as the work required for separating two phases from each other and is dependent on the surface tension properties of each contacting material. So, this method involves the evaluation of adhesion at the macroscale, based on surface tension properties of both polymer and metal phases. Both methods reveal that the adhesion is increasing as more azochromophore was introduced in the system. The major difference between these two methods is that the AFM force spectroscopy allowed for the determination of local adhesion properties, for example, at the bottom of SRG (the lowest height zones) and on the top of SRG (the highest height zones), while the contact angle assesses the changes in surface dispersive/polar character over a wide surface area (containing a few SRGs).

## 4. Conclusions

Polyimide supramolecular systems containing different amounts of azochromophore in the polyamidic acid were obtained in the form of flexible films by the thermal imidization process and were tested as supports for deposition of conductive metals in the fabrication of sensors for monitoring the temperature changes. The interface of the flexible polyimide part of the sensor was designed by means of UV-laser irradiation through a phase mask with 1000 grooves/mm, using a constant laser fluence of 45 mJ/cm^2^, a total delivered laser pulses of 300, and a pulse repetition frequency of 10 Hz. AFM studies revealed that the increase of the molar ratio of the azochromophore in the polyimide supramolecular systems improved the SRG’s quality, increased their heights to about 160 nm, and their periodicity to 70%, the bearing capacity of the surface, the fluid retention on the core and valley regions of the relief, and the surface anisotropy until the texture direction index reaches almost 0.200. These characteristics ensure the improvement of the sensitivity, accuracy, and response time of the device. To evaluate the compatibility between the flexible substrate and the inorganic layer, the AFM tips were modified by a silver sputtering process, the best result being obtained when the treatment time was 15 min. After the blind tip reconstruction was applied, and the quantitative characteristics of the cantilever were calculated, the force curve spectroscopy was used to evaluate the local nanomechanical properties. In this way, it was found that the average adhesion force between the silver-coated cantilever’s tip and the azo-polyimide substrate increased as the azo-dye quantity was higher, being 47 nN for Az_0.25_PSS-L, 73 nN for Az_0.50_PSS-L, 94 nN for Az_0.75_PSS-L, and 150 nN for Az_1.00_PSS-L. The supplementary wettability study sustained the AFM results, and the work of the adhesion of the textured polyimide surface with silver being the highest also for Az_1.00_PSS-L. These results indicated better interfacial compatibility of the flexible substrate of polyimide with the inorganic layer (silver) when the azo component and the polyamidic acid are in equimolar quantities. Such aspects are favorable for sensor fabrication, owing to the fact that they contribute to the better reliability of the device.

## Figures and Tables

**Table 1:** Amplitude and spatial parameters of the AFM investigated surfaces and the SRGs characteristics.

Sample	SRGs Characteristics				Amplitude and Spatial Parameters	
	H_SRG_ (nm)	Ra (nm)	Periodicity (%)	Period (μm)	Sq (nm)	Stdi
Az_0.25_PSS-L	52 ± 5	11.4	45.97	1.636	18.5	0.472
Az_0.50_PSS-L	90 ± 8	6.6	49.26	1.012	29.6	0.345
Az_0.75_PSS-L	125 ± 7	6.5	59.79	1.332	40.4	0.318
Az_1.00_PSS-L	158 ± 8	3.3	69.36	1.019	44.6	0.236

H_SRG_: average SRGs height; Ra: average roughness on top of SRGs; Sq: root mean square height of the surface; Stdi: texture direction index of the surface.

**Table 2:** Functional indexes and functional volume parameters evaluated from the Abbott curves.

Sample	Functional Indexes	Functional Volume Parameters			
	Sbi	Vmp (nm^3^/nm^2^)	Vmc (nm^3^/nm^2^)	Vvc (nm^3^/nm^2^)	Vvv (nm^3^/nm^2^)
Az_0.25_PSS-L	0.221	1.52	11.80	27.00	1.23
Az_0.50_PSS-L	0.320	1.67	23.30	43.80	2.14
Az_0.75_PSS-L	0.584	1.69	36.60	52.30	3.86
Az_1.00_PSS-L	1.053	0.68	42.60	52.30	4.41

Sbi: surface bearing index; Vmp: peak material volume; Vmc: core material volume; Vvc: core void volume; Vvv: valley void volume.

**Table 3:** The results of the contact angle, dispersive surface tension, polar surface tension, and work of adhesion between the samples and silver.

Sample Code	Contact Angle, °		$\sigma_{s}^{d}$, mN/m	$\sigma_{s}^{p}$, mN/m	$W_{Ad}$, mN/m
	Water	Formamide			
Az_0.25_PSS	77	57	26.94	8.18	78.17
Az_0.50_PSS	74	55	26.06	10.09	78.39
Az_0.75_PSS	69	50	26.61	12.67	80.64
Az_1.00_PSS	63	44	26.83	16.29	82.88
Az_0.25_PSS-L	73	54	26.21	10.55	78.87
Az_0.50_PSS-L	69	51	25.39	13.33	79.44
Az_0.75_PSS-L	62	44	25.83	17.44	82.20
Az_1.00_PSS-L	58	40	25.85	20.12	83.49

## Data Availability

Data sharing is not applicable to this article.

## Appendix A

The Abbott curve, presented in Figure A1, which is also called the surface bearing area ratio curve, is in general calculated by accumulation of the height distribution histogram and subsequent inversion. Dashed horizontal lines drawn through the Abbott curve (Figure A1) are marked with H1 at the intersection with mr1 line at 10% material ratio value and H2 at the intersection with mr2 line at 80% material ratio value. In this manner, three areas were created: the peak zone, the core zone and the valley zone. In the representative Abbott curve shown in Figure A1, the region of the curve colored in dark orange represent the material located in the peak zone, the one painted in light orange represent the material located in the core zone, the one colored in light blue represent the air located in the core zone and the one painted in dark blue represent the material located in the valley zone. The functional index (surface bearing index—Sbi) and the volume functional parameters (peak material volume—Vmp, core material volume—Vmc, core void volume—Vvc and valley void volume—Vvv) are defined from the Abbott curve, with respect to two previously mentioned bearing ratio thresholds, set by default to 10% (mr1) and 80% (mr2). Sbi characterizes the upper zone of the surface involved in wear phenomena and indicates the bearing property. Vmp is the volume of the material calculated from the Abbott curve, in the region above the dashed horizontal line H1, between 0% and mr1 (10%) bearing ratio. Vmc is the volume of material calculated from the Abbott curve, in the area between dashed horizontal line H1 and H2, between mr1 (10%) and mr2 (80%) bearing ratio. Vvc is the volume of air calculated from the Abbott curve, in the zone between the dashed horizontal line H1 and H2, between mr1 (10%) and mr2 (80%) bearing ratio. Vvv is the volume of air calculated from the Abbott curve, in the area below the dashed horizontal line H2, between mr2 (80%) and 100% bearing ratio. These parameters are expressed in units of volume per unit of surface (µm^3^/µm^2^ or nm^3^/nm^2^, depending on the heights of the surface features).
